# Oxidative Damage Under Microgravity Conditions: Response Mechanisms, Monitoring Methods and Countermeasures on Somatic and Germ Cells

**DOI:** 10.3390/ijms26104583

**Published:** 2025-05-10

**Authors:** Zekai Chen, Jingtong Xie, Chiyuan Ma, Pengfei Zhang, Xiaohua Lei

**Affiliations:** 1 Department of Biomedical Engineering, Southern University of Science and Technology, Shenzhen 518055, China; zk.chen@siat.ac.cn; 2 Center for Energy Metabolism and Reproduction, Shenzhen Institute of Advanced Technology, Chinese Academy of Sciences, Shenzhen 518055, China; jt.xie@siat.ac.cn (J.X.); cy.ma1@siat.ac.cn (C.M.); 3 Guangdong Key Laboratory of Nanomedicine, CAS-HK Joint Lab of Biomaterials, CAS Key Laboratory of Biomedical Imaging Science and System, Institute of Biomedicine and Biotechnology, Shenzhen Institutes of Advanced Technology, Chinese Academy of Sciences, Shenzhen 518055, China; pf.zhang@siat.ac.cn

**Keywords:** microgravity, oxidative stress, germ cell, ROS, antioxidant

## Abstract

With the growing human interest in space exploration, understanding the oxidative damage effects of microgravity on somatic and germ cells and their underlying mechanisms has become a pivotal scientific challenge for ensuring reproductive health during long-term space missions. In this review, we comprehensively summarize the molecular mechanisms of microgravity-induced oxidative stress, advanced detection methods, and potential protective strategies for germ cells. The evidence demonstrates that microgravity substantially compromises germ cell viability and embryonic developmental potential by disrupting mitochondrial function, increasing reactive oxygen species (ROS) production, and impairing antioxidant defenses. These alterations result in DNA damage, lipid peroxidation, and protein oxidation, thereby affecting cellular integrity and functionality. Furthermore, we discuss how cells respond to microgravity-induced oxidative stress through adaptive mechanisms, such as autophagy, apoptosis, and antioxidant systems, although these responses can have both beneficial and detrimental effects on cellular homeostasis. Additionally, this paper highlights the utility of fluorescent probes for detecting ROS levels under microgravity conditions, which are convenient and practical, but may require further optimization to improve sensitivity and specificity. To counteract these challenges, interventions such as antioxidants and artificial gravity systems show promise but need rigorous validation in prolonged microgravity environments. Finally, future research should integrate multi-omics approaches to unravel the oxidative damage network, advance space-adapted reproductive technologies, and provide essential theoretical insights and technical support for maintaining human reproductive health beyond Earth.

## 1. Introduction

As human scientific and technological endeavors advance, our gaze increasingly extends beyond Earth toward the vast and intriguing realm of space. However, the space environment presents significant differences and challenges compared to that of Earth, posing unique risks to human health. Among these challenges, the microgravity environment stands out as a critical factor requiring in-depth investigation. Microgravity refers to an environment where the acceleration caused by gravity or other external forces does not exceed 10^−5^ g [1]. In this unique environment, the effective gravitational force experienced by the human body is extremely low—only one-millionth or less of the gravity on the Earth’s surface. In a microgravity environment, objects appear “weightless” because the gravitational forces acting upon them are effectively canceled by other forces, leaving them in a state of approximate force equilibrium.

### 1.1. Damage to Somatic Cells from Different Systems in Microgravity

NASA’s twin space experiment profoundly illustrates the many adverse effects on astronauts’ health, and provides important science for future long-duration space missions [2]. In the microgravity environment of space, several human physiological systems are affected, including the skeletal system, the muscular system, and the immune system. In the skeletal system, the change in gravity results in the bones not needing to bear weight and the rapid loss of calcium from the bones [3]. On a cellular level, microgravity inhibits osteoblast function, while osteoclasts are relatively more active and their mediated bone resorption is enhanced [4,5]. For the muscular system, a prolonged microgravity environment can lead to muscle atrophy and loss of muscle mass [6]. This may involve complex mechanisms of microgravity on aspects of muscle energy metabolism and protein synthesis [7]. Microgravity has an equally wide-ranging effect on the immune system. Both naïve B cells and memory B cells exhibit reduced abundance under microgravity conditions [8]. Expression of CD86, a macrophage co-stimulatory molecule, is upregulated, and expression of M2 type macrophage-specific genes, such as Arg1, is also altered [9]. These changes may influence macrophage polarization, inflammatory responses, and overall immune function. Thus, human exploration in space is severely tested by the various health impairments associated with microgravity; however, there is a wealth of research that exposes the biology of these changes, and the mechanisms are relatively well understood.

### 1.2. Damage of Germ Cells in Microgravity

Unlike somatic cells, germ cells have a special significance in that changes in germ cells may be passed on to future generations. Overall, microgravity damages different types of germ cells. Microgravity causes a reduction in the number of spermatozoa and a decrease in their viability [10]. Other cells in the testes, apart from sperm, such as the Leydig cell, are also severely affected [11]. The oocyte quality is usually reduced in microgravity, and tends to interfere with the fertilization process [12]. Embryonic stem cells, on the other hand, change the direction of differentiation in microgravity and have increased apoptosis [13,14].

### 1.3. Methods of Simulated Microgravity

**Figure 1:**
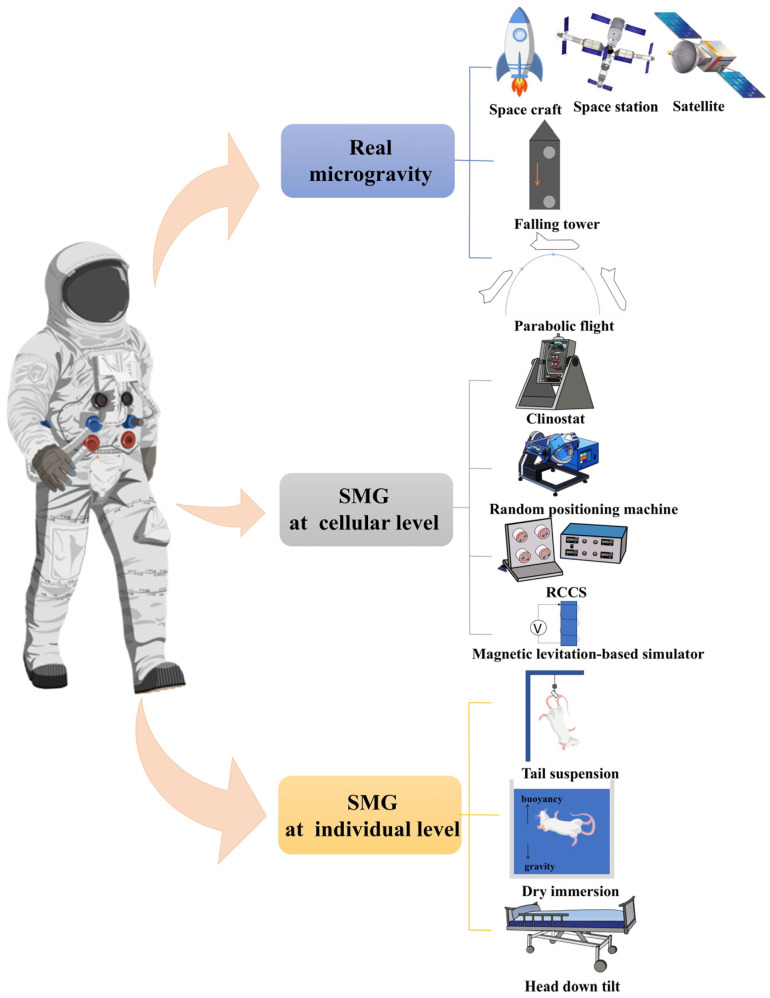
Various methods of SMG effects, divided into three categories: real microgravity, SMG at cellular-level, SMG at organism-level.

To explore the mysteries of the impact of microgravity on living organisms, it is essential for humanity to simulate the microgravity environment on Earth to study their effects and develop appropriate countermeasures. Currently, there are three primary categories of simulated microgravity (SMG) effects methods (Figure 1).

#### 1.3.1. Real (Simulated) Microgravity Environment

The first category involves experiments conducted in actual space environment. These can be achieved through missions aboard satellites [15], spacecraft [16], or at space stations, where researchers directly experience microgravity. The falling tower method simulates microgravity by allowing an object to undergo a free-fall motion within a controlled tower structure. Parabolic flight, another widely used method, involves placing individuals or organisms in an aircraft that follows a parabolic trajectory. Upon reaching its apex, the aircraft enters a downward dive, creating 20–40 s of time for SMG. Different realistic SMG methods have their own advantages and disadvantages for germ cell research. Satellites, spacecraft, or space stations provide a realistic long-term SMG environment, which is suitable for experiments that require long-term observations, such as germ cell development [17], but the disadvantage is that they are extremely costly. By contrast, parabolic flight and drop tower provide a shorter period of time and are suitable for short-term physiological changes in germ cells [18]. The drop tower method is the least expensive of these methods, but it is difficult to recover samples.

#### 1.3.2. Cellular-Level Simulation Methods

The second category focuses on cellular-level simulations. A clinostat is an important tool for studying the effects of microgravity on cells. Cells are loaded onto a clinostat in the petri dish, and the rotary motion of a clinostat mimics microgravity conditions. Depending on the number of rotating axes, clinostats can be divided into two-dimensional (2D) and three-dimensional (3D) variants. While 2D clinostats rotate around a single axis, 3D clinostats enable multi-axes rotation, providing a more comprehensive simulation. For example, random positioning machines (RPM) simulate microgravity by moving cells randomly in three dimensions [19]. Another approach is the rotary cell culture system (RCCS), developed by NASA, which induces a “weightless” state in cells by rotating the culture vessel horizontally [20,21]. A magnetic levitation-based simulator (MLS) represents another method, leveraging a non-uniform magnetic field to counteract gravitational forces acting on cells. For cell-level simulations, the clinostat is the least difficult to operate, while the MLS is the most difficult to implement and the most expensive to maintain. For germ cell culture, RCCS may be the most suitable choice, with moderate cost, little difficulty in operation, and precise control of various cell culture conditions [22], without the interference of shear stress affecting the germ cells, as is the case with RPM [23].

#### 1.3.3. Organism-Level Simulation Methods

At the level of whole organisms, several methods exist for simulating microgravity. Mice, commonly used as experimental models, are often subjected to tail suspension, where they are hung by their tails to maintain their backs or hind legs off the ground, thereby achieving the conditions of a microgravity effect. Dry immersion (DI) is another valid ground-based model for simulation of the physiological effects of weightlessness for some systems, involving submersion of the subject in a liquid medium to offset gravity through buoyancy. Head down tilt (HDT) is yet another effective technique for simulating the effects of microgravity on humans. In HDT studies, subjects lie on a tilted bed with their head positioned approximately 6° below their feet, mimicking the redistribution of bodily fluids observed in microgravity. Among these methods, the tail suspension method is the optimal choice for studying the effects of microgravity on animal germ cells, as it supports long-term mechanism research and efficient sample acquisition. HDT is suitable for a short-term effect assessment in humans.

Mechanics plays a crucial role in biology. The structure and function of various parts of the human body depend heavily on mechanical forces, from organ formation and tissue integrity maintenance to signal transmission between cells. The mechanical effects brought about by the microgravity environment can reshape the human body in numerous ways.

### 1.4. Oxidative Stress (OS) in Microgravity

A microgravity environment leads to increased oxidative stress (OS) in the human body [24]. In a healthy human body, oxidative and antioxidant mechanisms maintain a dynamic balance. ROS, including hydrogen peroxide (H_2_O_2_), superoxide anion (O_2_^−^), hydroxyl radical (OH), ozone (O_3_), and singlet oxygen (^1^O_2_), play critical roles in regulating various signaling pathways, such as cell proliferation [25], differentiation [26], and apoptosis [27]. While moderate ROS levels are beneficial, excessive ROS production disrupts redox homeostasis, leading to cellular damage, signaling imbalance, disease development, cell death, and impaired immune function [28,29]. In the case of germ cells, OS leads to germ cell damage, which can affect reproductive health. Therefore, it is important to study OS and its potential cellular damage and health of germ cells to protect astronauts’ health and improve reproductive success in space. This article will focus on the OS induced in cells by the microgravity environment.

The existing studies have shown that microgravity conditions significantly induce oxidative stress, disrupt mitochondrial function, increase ROS production, and impair antioxidant defense mechanisms. These changes lead to DNA damage, lipid peroxidation, and protein oxidation, thereby affecting cellular integrity and function. In addition, there is some understanding of how cells respond to microgravity-induced oxidative stress through adaptive mechanisms, such as autophagy, apoptosis, and antioxidant systems. Although some progress has been made, more studies on the specific molecular mechanisms of cellular oxidative damage under microgravity conditions, effective monitoring methods, and practical protective strategies have been conducted from the perspective of somatic cells and germ cells, as cells concerned with future human reproduction in space have not been studied much under this model, as well as how to effectively detect and implement protective measures in space. The research on how to effectively detect and implement protective measures in space is also far from sufficient. Given the long-term nature of space exploration and the importance of human reproductive health, we decided to write this review. We attempt to provide theoretical support and technical guidance for future research on reproductive medicine in space by comprehensively summarizing the molecular mechanisms of microgravity-induced oxidative stress in somatic and germ cells, advanced detection methods, and potential protective strategies.

## 2. Effect of Microgravity on Cellular Physiology

**Figure 2:**
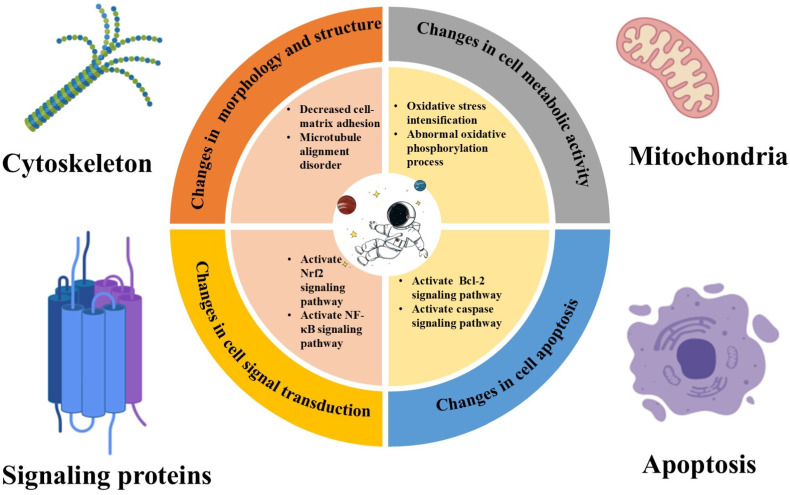
The effects of microgravity on various aspects of cells. It can cause changes in the following four areas: cell morphology and cytoskeleton, cell metabolic activity, cell signal transduction, and cell apoptosis.

Microgravity profoundly alters the cellular environment at both mechanical and physiological levels [30], significantly impacting cellular processes (Figure 2). These effects are mainly reflected in changes in gene expression, protein synthesis, and metabolic pathways, in addition to the generation of ROS from multiple pathways.

### 2.1. Microgravity’s Influence on Cellular Gene Expression

Microgravity induces widespread alterations in gene expression across various somatic cell types. For instance, immune cells experience dramatic shifts in their number and subpopulation compositions under microgravity conditions [31], which in turn affect their gene expression profiles. Early studies demonstrated that the induction of nearly 100 genes is suppressed under microgravity [32]. The regulation of these genes is primarily mediated by key transcription factors NF-kappaB, CREB, ELK, AP-1, and STAT, which play central roles in T cell activation, proliferation, differentiation, and function. This suggests that microgravity has an inhibitory effect on both T cell development and function. Subsequent studies have corroborated this observation. Martinez et al. [33] reported that the gene expression of SLAMF1 and Iigp1, which are critical for T cell activation and function, is downregulated under microgravity. Collectively, the effects of the microgravity on gene expression extend beyond immune cells, affecting a board range of cell types.

Microgravity induces widespread alterations in gene expression across various cell types. Similarly, there is substantial evidence that microgravity affects gene expression in germ cells. Microgravity causes germ cells to express different levels of skeleton-related genes than on ground level. Sperm genes encoding actin and actin-binding proteins are methylated in microgravity [34]. Numerous mRNAs encoding skeleton-associated proteins were also found to have much increased expression in the oocytes [35], and this skeleton reorganization may be related to the reproductive function of the oocytes. For embryonic stem cells, some genes associated with differentiation are also disturbed by microgravity [36,37]. Changes in other related genes will be discussed later in the article, including oxidative stress-related genes, as well as changes in the expression of apoptosis-related genes, heat stress genes, and autophagy-related genes in germ cells under the influence of microgravity.

### 2.2. Microgravity’s Influence on Cellular Protein Synthesis

Protein synthesis represents a direct outcome of gene expression, and is therefore naturally regulated by microgravity. The effect of microgravity on cellular protein expression is multifaceted, influencing cellular morphology, function, and metabolism at multiple levels. A notable effect is the alteration of cytoskeleton-associated proteins. Modification of the cytoskeleton of TCam-2 cells in microgravity [38], and cytoskeletal proteins such as Lys9 were shown to be upregulated in expression in the ovaries [39].

Mitochondria, which are essential for fat, protein, and carbohydrate metabolism, also exhibit functional adaptations under microgravity. Previous studies have demonstrated that components of the mitochondrial electron transport chain (ETC) are differentially regulated. The expression of mitochondrial complex II was downregulated by 50%, complex IV was downregulated by about 14%, while complex III was increased by 60% [40,41]. The changes in the components of the respiratory chain may be related to the increased abundance of mortalin and AFG3L2 [42], which are crucial for the assembly and function of the mitochondrial complexes. For germ cells, it has been shown that microgravity causes a decrease in cytochrome c-1 expression in spermatozoa [10].

The tricarboxylic acid (TCA) cycle, a fundamental pathway for energy metabolism, is similarly affected by microgravity. Enzyme activities within the TCA cycle are broadly downregulated, potentially due to the lower turnover rates observed during spaceflight [43]. This reduction in enzyme activity also impacts related pathways, including gluconeogenesis and the glyoxylate cycles. In summary, microgravity modulates cellular protein expression, thereby influencing cellular morphology, function, and metabolic activities at multiple levels.

### 2.3. Microgravity’s Influence on Cellular Metabolic Pathways

Microgravity has a wide range of effects on cellular metabolism, affecting amino acid metabolism [44], glucose metabolism [45], and lipid metabolism [46]. In addition to this, microgravity affects mitochondrial function and energy metabolism, often leading to a slowdown in the metabolic activity of cells. Microgravity also increases the production of cellular ROS, leading to a range of biological effects that have potentially negative impacts on human health. This will be further discussed in the following sections.

### 2.4. Microgravity’s Influence on the Mechanisms of ROS Generation

#### 2.4.1. Mitochondrial Dysfunction

In microgravity, numerous potential pathways for ROS generation exist, and the underlying mechanisms are very complex. The ETC is the primary source for ROS production [47]; however, in microgravity, the mitochondrial function tends to become impaired. Dysfunctional mitochondria generate elevated levels of ROS, which, in turn, exacerbate mitochondrial damage through a self-perpetuating feedback loop. The mechanisms by which mitochondrial dysfunction lead to ROS generation are multifaceted and intricate.

Microgravity may interfere with ETC function via several pathways. As previously noted, the microgravity environment induces alterations in various complexes within the ETC [40], leading to impaired ETC function. This impairment results in electron leakage, and the leaked electrons contribute to ROS production. Additionally, the reduction in membrane potential (ΔΨm) on both sides of the inner mitochondrial membrane under microgravity likewise promotes the production of ROS, such as O_2_^−^ [48].

Piezo1, a critical mechanosensory protein in cellular ion channels, plays an important role in mechanical response [49]. Altered mechanosignaling due to microgravity might promote Piezo1 activation. It has been shown that Piezo1 activation leads to an increase in intracellular calcium ion concentration, which in turn activates mitochondrial fission, and triggers mitochondrial dysfunction via the Ca^2+^/CaMKII/Drp1 axis [50]. However, silencing Piezo1 improves mitochondrial structure and function, which rescues CEP cell senescence and apoptosis in inflammatory conditions.

#### 2.4.2. Altered Enzyme Activity

The microgravity environment also influences ROS production by modulating the activity of various enzymes. NADPH oxidase generates ROS primarily through catalyzing reactions, and the microgravity environment has been shown to elevate NADPH oxidase activity, thereby increasing ROS production [40]. At the same time, SMG also reduced ATPase activity, leading to a decrease in intracellular ATP synthesis, which in turn led to an increase in the activity of cytochrome c oxidase (CytOx), a key regulator of inhibitory mitochondrial function, resulting in a surge in ROS production [51].

Key enzymes in the TCA cycle, such as succinate dehydrogenase (SDH), fumonisinase (FUMH), and malate dehydrogenase (MHDM), exhibit reduced activity under microgravity conditions [43]. This disruption impairs the TCA cycle, leading to a large accumulation of succinate within the cell. Elevated succinate levels enable reverse electron transport to complex I of ETC [52], resulting in the formation of substantial amounts of the ROS.

Additionally, the activity of certain antioxidant enzymes may be inhibited under microgravity, contributing to excessive ROS generation. However, this situation is not absolute, and will be further discussed in the section on antioxidant enzymes. In summary, the increase in ROS production observed under microgravity may result from a combination of changes in the activity of various enzymes.

## 3. Oxidative Damage to Cells in Microgravity

### 3.1. Direct Damaging Effects of ROS on Germ Cells

Germ cells are a vital aspect of space research because understanding reproductive health, especially sperm, oocytes, and early embryos, is essential for future human reproduction in space as well as even space migration. Germ cells are particularly vulnerable to high levels of the ROS, which can impair their function and affect embryo development [53,54]. These effects may manifest as DNA damage, lipid peroxidation, and protein modification.

#### 3.1.1. DNA Damage

Microgravity-induced OS has been shown to affect a wide range of cells [55], and excessive ROS can induce damage to the DNA of germ cells. In spermatozoa, low levels of ROS are involved in several physiological processes, such as energy acquisition, signaling, and acrosome reaction [56,57]. Excessively high ROS can lead to DNA damage in spermatozoa [58,59]. ROS directly modify DNA, producing oxidized products, such as 8-hydroxy-2-deoxyguanosine (8-OHdG) [60], which has been shown to cause DNA damage. Additionally, ROS can induce DNA cross-linking and DNA strand breaks [61]. In embryonic stem cells, it has been shown that excess ROS can damage DNA [62]. High levels of ROS are also a critical factor contributing to DNA damage in the oocytes [63]. Elevated ROS levels lead to mutation and damage in the oocyte mitochondrial DNA (mtDNA). Research has shown that maintaining the mtDNA copy number can mitigate ROS-induced oocytopenia and female reproductive aging [64]. However, the effects of microgravity on germ cell DNA are controversial. Take male germ cells as an example, microgravity causes epigenetic changes in mouse spermatozoa [65]. However, it has also been found that microgravity induces a final round of DNA replication in Kit-positive spermatogonia [22]. This exemplifies the complex mechanisms by which microgravity affects the DNA of germ cells in addition to OS, and requires subsequent more in-depth studies.

#### 3.1.2. Lipid Peroxidation

Lipid peroxidation refers to the formation of peroxides from unsaturated fatty acids in response to oxidation, a process that can cause damage to lipid structures in cells such as cell membranes [66]. The sperm plasma membrane, an essential outer structure of spermatozoa, plays an important role in protecting sperm, regulating motility, participating in sperm-egg recognition and conjugation, enabling the acrosome reaction, as well as sperm maturation and capacitation. Polyunsaturated fatty acids (PUFA) are the major lipid components of the sperm plasma membrane, and when high levels of ROS are present, the sperm lipid peroxidation process involving PUFA is mainly presented as a cascade reaction [67]. It can be summarized that hydrogen atoms isolated from PUFA in the sperm plasma membrane and free ·OH can form lipid radicals, which can further react with oxygen to generate peroxyl radicals, which tend to react further with hydrogen atoms in PUFA, which leads to the continuous formation of lipid radicals and lipid peroxidation. The cascade reaction ends with the formation of lipid aldehydes, such as malondialdehyde (MDA) and 4-hydroxynonenal (4-HNE).

In addition to damaging the sperm plasma membrane, lipid peroxidation can also damage sperm in another way. The electrophilic nature of lipid aldehydes, particularly 4-HNE, causes extensive damage to spermatozoa. For instance, 4-HNE covalently binds to proteins through Michael addition reactions, forming protein adducts that impair sperm function [68]. Elevated MDA levels correlate negatively with sperm viability, survival, and acrosomal enzyme activity. Similarly, OS-induced lipid peroxidation damages the oocytes, where 4-HNE disrupts meiosis and causes chromosome misalignment [69].

#### 3.1.3. Protein Modification

High levels of ROS induce protein modification in germ cells, leading to multifaceted dysfunction. In spermatozoa, the production of 4-HNE may also lead to sperm protein oxidation. SDHA is a subunit of SDH complex II. Aitken et al. found that 4-HNE covalently binds to SDHA, causing it to auto-oxidize, and after oxidation its electrons are transferred to oxygen molecules, which in turn impairs mitochondrial function and induces apoptosis in spermatozoa [68]. Excess ROS also alter thiol/disulfide pairs in sperm proteins, affecting the structure and function of critical proteins [70]. As an ROS-dependent protein modification, S-glutathionylation may also impair sperm function [71]. Excess ROS compromise these defenses, further exacerbating oxidative stress.

In the oocyte, excess ROS mediate the meiotic process, leading to oxidation of spindle microtubule proteins, ultimately resulting in aneuploid embryos [72]. Glutathione (GSH) protects the spindle structure by maintaining the sulfhydryl-reduced state during oocyte maturation. Elevated ROS inhibit the expression of enzymes related to GSH synthesis, diminishing its protective effect on the oocytes and leading to impaired maturation and reduced fertilization rates. The phosphorylation level of MAPK, an essential enzyme in spindle assembly in the oocytes, is also subject to inhibition by high levels of the ROS [73].

### 3.2. Somatic Cell Antioxidant Enzyme Systems in Microgravity

Under the tough antioxidant challenge, antioxidant enzymes have been shown to exhibit remarkable resistance to excessive oxidation through complex mechanisms. Antioxidant enzymes play a key role in a biological organism’s defense against OS, and are the primary tools for cellular scavenging of the ROS. Notable antioxidant enzymes include superoxide dismutase (SOD), catalase (CAT), and glutathione peroxidase (GPx).

#### 3.2.1. Superoxide Dismutase

**Figure 3:**
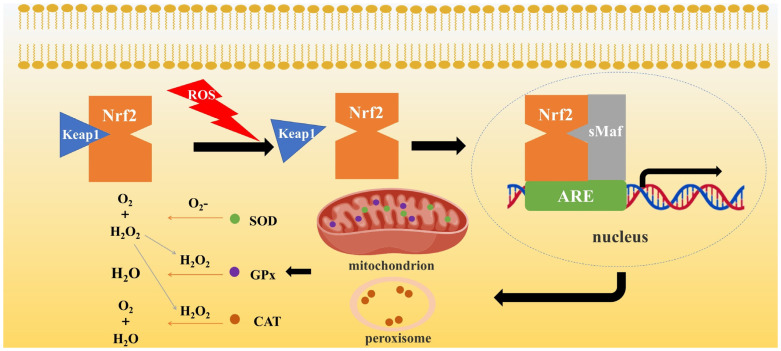
Activation of the Nrf2/ARE signaling pathway in response to OS in cells and its antioxidant effects. Nrf2 binds to Kelch-1ike ECH-associated protein 1 (Keap1) in normal physiological conditions. Upon cellular exposure to OS, Nrf2 dissociates from Keap1 and is activated. Activated Nrf2 enters the nucleus and dimerizes with sMaf, and the dimer subsequently binds to the antioxidant response element (ARE), promoting the expression of antioxidant genes in the cell. This leads to a rise in the expression of SOD and GPx in the mitochondria, and CAT in the peroxisome, which work together to counteract intracellular OS. SOD can catabolize O_2_^−^ into oxygen and H_2_O_2_, and the catabolized H_2_O_2_ can be further catalyzed by GPx to produce water, or by CAT to water and oxygen, as well.

Superoxide dismutase (SOD) is a pivotal antioxidant enzyme that is found in abundance in animals, plants, and microorganisms. O_2_^−^ can damage cell structure and function by damaging DNA, causing lipid peroxidation, and oxidizing iron–sulfur clusters of proteins. Under the catalytic action of SOD, O_2_^−^ undergoes disproportionation and is converted to H_2_O_2_ and O_2_, thus ROS levels decrease. O_2_^−^ has numerous sources, and SOD exhibits distinct subcellular localizations [74], allowing for precise, localized control of ROS signaling. Oxidative stress (OS) activates the upregulation of SOD, which is mainly achieved through the Nrf2/ARE signaling pathway. In addition to CAT and GPx, which are also located downstream of the Nrf2/ARE signaling pathway and are regulated there (Figure 3), OS has been shown to induce the transfer of Nrf2 to the antioxidant response element (ARE) that binds to the promoter region of the SOD gene in the nucleus, thereby promoting the transcription of the antioxidant gene and reducing the damage caused by biological OS.

SOD has been demonstrated to play a critical role not only in limiting the oxidative toxicity of O_2_^−^ but in regulating ROS signaling [75]. SOD1, by catalyzing the conversion of O_2_^−^ to H_2_O_2_, can affect the activity of protein tyrosine phosphatases (PTPs). PTPs play a critical role in cellular signaling, and the exertion of their activity is dependent on cysteine residues on PTPs [76], whereas H_2_O_2_-mediated oxidation of cysteines leads to the loss of PTPs activity. Additionally, SOD has been observed to regulate the NF-κB signaling pathway by modulating the levels of the ROS, which, in turn, impacts the process of apoptosis [77]. Furthermore, SOD has been shown to influence the activity of the AP-1 and JAK–STAT signaling pathways, thereby affecting cell growth and apoptosis [78,79].

#### 3.2.2. Catalase

H_2_O_2_ has been considered more cytotoxic than O_2_^−^ due to its ability to penetrate most cell membranes. CAT is an antioxidant enzyme found in almost all living organisms. The primary function of CAT is to remove H_2_O_2_, a process that involves the catalytic decomposition of H_2_O_2_ into water and oxygen [80]. The processing of O_2_^−^ by SOD also leads to the subsequent treatment of H_2_O_2_ by CAT, illustrating the collaborative interaction between different antioxidant enzymes. H_2_O_2_ obtained after SOD processing of O_2_^−^ is also descended to be further processed by CAT, which also reflects the collaboration between different antioxidant enzymes. Similar to SOD, OS also leads to upregulation of CAT expression mainly through activation of the Nrf2/ARE signaling pathway [81].

#### 3.2.3. Glutathione Peroxidase

Glutathione peroxidase (GPx) is a family of crucial intracellular antioxidant enzymes that play a critical role in the regulation of redox homeostasis. The GPx family is currently known to have eight members. The selenium atom in selenocysteine in GPx1–4 and GPx6 is able to accept electrons from GSH, generate GSSG, and convert peroxides to non-toxic water or alcohols [82]. Notably, GPx5 plays a pivotal role in safeguarding spermatozoa against OS [83], while GPx7 facilitates the transduction and release of OS signals through its interaction with glucose-regulated protein and protein disulfide isomerase [84]. A similar interaction between GPx8 and PDI has been observed, leading to the attenuation of OS in the endoplasmic reticulum and the delay of cellular senescence [85]. The activity of GPx is also influenced by the levels of NADPH, and decreased levels of NADPH result in impaired activity.

#### 3.2.4. Effect of Microgravity on Antioxidant Enzymes

The microgravity environment exerts a significant influence on antioxidant enzyme activity, though the underlying mechanisms are intricate. A study of eight astronauts aboard the International Space Station revealed a decline in SOD activity within their erythrocytes following antioxidant testing [86]. In a separate study, Wang et al. [87] exposed PC12 cells to SMG to investigate the alterations in antioxidant enzymes. Their findings indicated that, after a 12-h exposure to SMG, the activities of SOD, CAT, and GPx increased. However, by 96 h, a decrease in enzyme activity was observed. This may be due to a severe imbalance in intracellular oxidative homeostasis caused by high levels of OS leading to reduced enzyme activity. In a separate study, Mao et al. investigated oxidative damage in mouse brains under SMG by means of anti-orthostatic tail suspension [88]. SOD expression levels were reduced in the experimental group compared with the control group.

Germ cells have a variety of the same antioxidant enzymes as somatic cells, and the mechanisms are congruent. There are also few reports on the antioxidant enzyme activities of cells related to the reproductive system under microgravity, but the results were not trending either. In TCam-2 cell samples exposed to SMG, not only was the GPX1 expression reduced but the NCF1 gene expression level was also reduced, and the SOD, XDH, CYBA, NCF-2, TXN, and TXNRD genes were not affected [89]. However, a contradictory report indicated that Moustafa [90] simulated OS on the testes of mice exposed to SMG, and the testes showed increased levels of SOD, CAT, GPx, and total antioxidants. It has been found that glutathione levels and antioxidant enzyme activities were increased in the treated embryos under SMG [91]. The effects of microgravity on antioxidant enzyme levels have shown conflicting results in different studies, which may be due to a variety of factors. These include the experimental model, SMG simulation conditions, and exposure time. Tissue specificity is also a salient factor; different organs or tissues may exhibit varied responses to OS, such as reproductive organs and the brain. Furthermore, cells may initially experience elevated antioxidant enzyme levels to counteract OS during the initial phase of microgravity treatment. However, subsequent phases may result in a gradual depletion of the antioxidant system, leading to a subsequent decrease in antioxidant enzyme levels.

### 3.3. Cell Fate Reprogramming Triggered by Oxidative Stress in Microgravity

#### 3.3.1. Apoptosis in Germ Cells

Apoptosis refers to the programmed death of cells, and OS can induce apoptosis through various signaling pathways. OS can induce apoptosis through activation of caspase family proteins and induction of mitochondrial dysfunction, among other activities. Li et al. [92] found significant apoptosis of mouse spermatozoa under SMG and an increase in p53 and Bax protein expression. The p53 and Bax proteins play a crucial role in the apoptosis signaling pathway. A follow-up study used the same tail-hanging suspension method and immunohistochemical staining of the p53 protein in testicular tissues of mice in the experimental group. The results showed a significant increase in the death index, a significant increase in p53 protein expression, and more intense staining of cell nuclei in the tail suspension group of mice [93]. Similarly, the oocytes showed a greater number of apoptotic deaths in microgravity compared to normal gravity. For embryos, the microgravity environment significantly leads to apoptosis of embryonic cells. Wang et al. [13] found that SMG caused increased apoptosis and impaired DNA repair in mouse embryonic stem cells (mESC), and Cao et al. [94] used the rotating wall vessel bioreactor (RWVB) to simulate the effects of SMG on the pre-implantation embryonic development in mice, finding that embryonic development was significantly impaired and the number of apoptotic cells increased. Another study showed that SMG caused apoptosis in E2.5 mice and in E3.5 embryos, which may be related to the SAPK/JNK signaling pathway [17]. OS-induced apoptosis in microgravity has multiple significant effects on germ cells. Apoptosis can have a positive effect on reproductive health by preventing germ cells with severe DNA damage from passing on faulty genetic information to their offspring. However, the massive death of germ cells brought about by excessive OS will also impair the body’s reproductive function and may lead to diseases such as teratozoospermia [95].

#### 3.3.2. Autophagy in Somatic Cells and Germ Cells

Autophagy is the process by which lysosomes and autolysosomes degrade and recycle damaged organelles and denatured proteins in cells. Experiments have shown that microgravity induces the expression of autophagy-related genes and proteins in other cells. In mouse bone marrow-derived nonadherent cells cultured under SMG, the mRNA expression levels of autophagy-related genes Atg5, LC3, and Atg16L increased 20-fold, 35-fold, and 2.8-fold, respectively. Microgravity also significantly increased the expression of autophagy-related proteins. For instance, also under the above SMG conditions, the protein expression levels of Atg5 and LC3-II increased 8.0-fold and 7.0-fold, respectively [96]. It was also found that SMG can significantly increase the number of autophagic vesicles in cells [97].

Studies of microgravity-induced OS for germ cell autophagy are scarce, but Ferranti et al. [38] revealed that microgravity can induce autophagy in TCam-2 cells. This suggests that microgravity may also cause such adaptive changes in other germ cells on a mechanistic level. Based on this idea, we next address the effects of autophagy on germ cells, possibly caused by microgravity.

Autophagy plays a protective role in the normal physiological processes of germ cells. Specifically, in the context of spermatozoa, autophagy plays a pivotal role in numerous physiological processes, including spermatogenesis, the maintenance of normal sperm morphology, and the acrosome reaction. Autophagy activation has been shown to remove the ROS within a certain range, thereby maintaining the normal function of spermatozoa [98]. However, autophagy is a “double-edged sword”. Autophagy can aid in combating OS in germ cells; however, it can also be hindered under conditions of excessive OS, leading to detrimental outcomes for germ cells. Elevated OS has been shown to exert a detrimental effect on sperm autophagy, with excessive ROS leading to a disruption in the intracellular redox balance. In this case, the autophagy process is inhibited [99], which seriously affects a variety of normal physiological processes mediated by autophagy, impairs sperm viability, and prevents it from removing damaged cellular components in a timely manner, ultimately leading to sperm damage or even death. Similarly for the oocytes, OS is one of the most important factors leading to the decrease in the oocyte quality, and high levels of OS may lead to the failure of intracellular antioxidant systems, inhibit autophagy processes, and even trigger apoptosis [100]. It is important to mention that OS causes both apoptosis and autophagy. These two processes are closely related, and autophagy is a protection for the cell under normal conditions, which maintains the survival of the cell and avoids its programmed apoptosis by removing damaged mitochondria and other organelles. However, it can also promote apoptosis under special circumstances, acting synergistically with apoptosis. The two also share many signaling pathways and proteins, such as Beclin 1, p53, and Bcl-2 family proteins [101]. Thus, autophagy and apoptosis under OS have complex interrelationships in germ cells, and these interactions involve multiple molecular mechanisms and signaling pathways. A deeper understanding of these relationships could provide a theoretical basis for new strategies to cope with OS.

Subsequent studies on germ cells should still pay attention to investigating the regulation of germ cell autophagy caused by OS induced by microgravity, since its excessive levels may lead to functional damage and death of germ cells, and may also seriously affect important reproductive processes such as fertilization.

#### 3.3.3. Cell Cycle Checkpoint Changed in Somatic Cells and Germ Cells

Cell cycle checkpoints are key mechanisms in the cell cycle regulatory system that monitor whether the intracellular environment and external conditions are appropriate for the smooth progression of the cell cycle. A very large number of studies have now demonstrated that OS significantly affects somatic cell cycle checkpoints, and that OS can influence the function of cell checkpoints through a variety of mechanisms [102]. It has been found that OS-induced DNA damage can activate cell cycle checkpoints through the activation of ataxia-telangiectasia mutated (ATM) and ataxia-telangiectasia and Rad3-related (ATR) kinases [103]. These kinases further phosphorylate downstream targets, such as p53, leading to cell cycle arrest. OS can also regulate cell cycle checkpoints by affecting the activity of cyclins and cell cycle protein-dependent kinases (CDKs), leading to cell cycle arrest.

For germ cells, cell cycle checkpoints are able to detect DNA damage and pause the cell cycle to repair it, thus avoiding passing on damaged DNA to offspring. The proper functioning of cell cycle checkpoints helps to maintain the quality and quantity of germ cells, ensuring the viability of the sperm and oocytes, and thus the quality of the embryos [104]. OS has a significant effect on cell cycle checkpoints in germ cells. OS leads to activation of the CHEK2 signaling pathway in the oocytes [105]. CHEK2 activates its downstream TAp63 and p53, leading to oocyte apoptosis. For spermatozoa, oxidative stress can cause DNA damage in spermatocytes, leading to the activation of p53. Expression of CDK inhibitors, such as p21 and p16, can be induced by p53. These inhibitors can bind to the CDK/cyclin complex and inhibit its activity, leading to sperm cell cycle arrest [106].

Similarly, it has been shown that microgravity-induced OS in germ cells is closely related to changes in cell cycle checkpoints. Morabito et al. [107] showed decreased cell proliferation rate, increased OS, and delayed cell cycle progression in TCam-2 cells after 24 h of exposure to SMG. This was manifested by a significant increase in the proportion of cells in the G0/G1 phase of the cell cycle. This indicates that the microgravity environment caused the cell cycle to stagnate in the G0/G1 phase, and the cells were unable to enter the S phase for DNA replication, which in turn affected the normal cell division and proliferation. They also found that the use of the antioxidant Trolox could counteract microgravity-induced cell cycle arrest, suggesting that OS is an important factor in microgravity’s influence on cell cycle checkpoints and that OS can be alleviated by antioxidants, thus restoring the normal progression of the cell cycle. In summary, microgravity-induced OS has a profound effect on cell cycle checkpoints in germ cells. It interferes with the normal function of cell cycle checkpoints through a series of complex mechanisms, such as activation of ATM and ATR kinases, and affecting the activity of cell cycle proteins and CDKs, which in turn leads to abnormal meiosis, genomic instability, and reproductive impairment in germ cells

### 3.4. Gene Expression Remodeling by OS in Microgravity

Microgravity-induced OS can lead to significant changes in somatic and germ cell gene expression, and this effect is multifaceted. Understanding these changes is critical to elucidate the mechanisms of microgravity-induced oxidative damage and to develop protective strategies.

#### 3.4.1. Nrf2-Related Signaling Pathway

For both somatic cells and germ cells, Nrf2 is a central transcription factor in the antioxidant defense system and regulates the expression of multiple antioxidant genes, including SOD, CAT, GPx, and NAD(P)H:quinone oxidoreductase 1 (NQO1) by binding to ARE [108], and thus the corresponding antioxidant mechanism to maintain oxidative homeostasis of cells. The specific mechanisms underlying this process have been previously delineated and will not be repeated here.

There is little information about the effect of OS caused by microgravity on the expression of the Nrf2 gene in germ cells. However, Nrf2 and the related signaling pathways are of great significance in the context of antioxidant effects in germ cells. The activation of the Nrf2 pathway can lead to a substantial reduction in oxidative damage to the mouse testes. Furthermore, the activation of the Nrf2 pathway was found to enhance the levels of CAT and SOD. The mRNA levels of the Nrf2-regulated genes Nrf2, HO-1, GCLC, and NQO1 exhibited a significant increase, and the expression of Nrf2 proteins in the nucleus also demonstrated upregulation. For the mouse uterus, the activation of Nrf2 has been shown to have an antioxidant effect, as indicated by an increase in the levels of antioxidant genes. Furthermore, the expression of caspase-3 and caspase-9 was reduced in the mouse uterine cells, suggesting that OS-induced activation of the Nrf2 pathway provides a protective effect against OS, prevents lipid peroxidation, and inhibits apoptosis [109].

In conclusion, Nrf2 is essential for shielding cells from oxidative damage brought on by microgravity. When it is activated, antioxidant genes are upregulated, strengthening the body’s defenses against oxidative damage.

#### 3.4.2. Heat Shock Proteins

Heat shock proteins (HSPs) are a widespread class of heat stress proteins found in mammals. The production of ROS leads to the trimerization and translocation of heat shock factor (HSF) from the cytoplasm to the nucleus, and its subsequent binding to the heat shock element (HSE) initiates the transcription of HSP genes. HSPs are of tremendous significance to the antioxidant system. Proteins in cells under OS are prone to misfolding, and this misfolding leads to changes in protein structure, which prevents them from performing their original functions. HSP70 and HSP90 in the heat shock protein family can act as molecular chaperones to help proteins fold correctly and prevent them from denaturing and inactivating under OS. HSP27 can enhance cellular antioxidant capacity by regulating the Nrf2 signaling pathway and increasing the expression of antioxidant enzymes. HSP70 can also enhance antioxidant effects by promoting the activity of GPx and GR [110]. For example, HSP27 protects cells from OS-induced apoptosis by inhibiting caspase-3 activity [111], while HSP70 reduces OS-induced apoptosis by inhibiting the JNK and p38 MAPK pathways [112].

Microgravity-induced OS may lead to upregulation of somatic cell heat shock proteins [113]. For germ cells, there have also been a number of studies describing the role of microgravity-induced OS on HSPs. OS was exacerbated in mouse spermatozoa, and a significant increase in HSP70 expression was observed in those exposed to SMG [90]. Shimada and Moorman [114] showed that zebrafish embryos showed an increase in mRNA expression of HSP70 under SMG. Radmanesh et al. found that OS significantly increased the expression of HSP 70-2a and HSP 90 in cells in mouse testis [115]. Upregulation of the expression of HSPs in germ cells can assist germ cells in counteracting a range of detrimental effects of OS brought about by microgravity.

#### 3.4.3. Apoptosis-Related Genes

For somatic cells, the Bcl-2 family of genes are genes closely related to the regulation of apoptosis. The Bcl-2 family of genes encodes both the pro-apoptotic Bax protein and the anti-apoptotic Bcl-2 protein, which together form the typical apoptotic pathway known as Bcl-2/Bax. Bax can form a dimer by itself or a heterodimer with Bcl-2. High Bax expression promotes apoptosis, whereas high Bcl-2 expression inhibits apoptosis. The Bcl-2/Bax pathway changes in microgravity-induced OS in germ cells have not been studied much; however, other models causing OS in germ cells are also informative. Zhang et al. [116] showed that spermatozoa from patients with oligospermia and asthenospermia showed multiple indicators of OS compared to spermatozoa from normal males, as well as demonstrating a marked Bax protein expression and a corresponding decrease in Bcl-2 protein expression. Another study showed that 4-nonylphenol induced oxidative damage in mouse spermatozoa [117], again with an upregulation of Bax and a downregulation of Bcl-2. A marked decrease in the Bcl-2/Bax ratio can also be observed in models of oxidative damage in the oocytes. For embryos [118], it has been shown that oxidative damage can reduce the Bcl-2/Bax ratio through the activation of JNK and ERK, leading to apoptosis in early embryos. In conclusion, Bcl-2/Bax levels are of important guidance in OS in germ cells, and a decrease in its ratio often leads to germ cell apoptosis, which is detrimental to reproductive health.

In addition to the above, OS can activate genes, such as p53, Fas and FasL, Caspase, and AIF, leading to germ cell apoptosis, which in turn affects reproductive function. These genes play an important role in OS-induced germ cell apoptosis and could be potential targets for the prevention and treatment of reproductive disorders in microgravity.

### 3.5. Risks to Other Aspects of the Reproductive System

In addition to the cellular level, OS resulting from microgravity may have profound effects on other levels of the reproductive system. The effects of OS on fertility and embryonic development are particularly notable in microgravity.

#### 3.5.1. Microgravity-Induced ROS on Female Reproductive System

In women, the ovary is a vital reproductive organ as well as a key location for the growth and generation of the oocytes [119]. The hypothalamic–pituitary–ovarian axis is an important regulator of the female reproductive endocrine system and plays an important role in regulating ovulation and fertility [120]. It has been demonstrated that the hypothalamus’s production of gonadotropin-releasing hormone (GnRH) is impacted by the microgravity environment [121]. In addition, microgravity-induced fluid shifts may affect the pituitary gland, resulting in decreased levels of GnRH, follicle-stimulating hormone (FSH), luteinizing hormone (LH), and testosterone. Similarly, many studies have shown that high levels of ROS can have an impact on the total function of organs on the axis, which may ultimately be detrimental to ovarian hormone level expression and follicular development, among other things. OS plays a significant role in the development of polycystic ovarian syndrome (PCOS), a prevalent reproductive endocrine illness in women with a complex pathophysiology [122]. Patients with PCOS have elevated levels of ROS in the follicular fluid and serum compared to healthy women [123], which affects follicular growth and maturation, and leads to a decrease in the quality of the oocytes, which can affect women’s reproductive health [124]. The corpus luteum is a physiological structure formed after ovulation that is essential for the female reproductive cycle. The corpus luteum secretes estrogen and progesterone, which synergize with each other to regulate the normal female menstrual cycle [125]. High levels of ROS can lead to luteal insufficiency and even infertility in women [126]. In summary, OS has multiple negative impacts on the health of the female reproductive system.

#### 3.5.2. Microgravity-Induced ROS on Male Reproductive System

For men, the hypothalamic–pituitary–gonadal axis is similarly important. The microgravity-induced ROS surge can affect the hypothalamic–pituitary–gonadal axis-mediated regulation of hormone levels [127], impairing male reproductive health. The male testes produce testosterone, and testosterone levels are critical for sperm quality. OS in the testes can damage mesenchymal stromal cells in the testes and affect their ability to synthesize testosterone [128]. Similarly, there has been substantial evidence that microgravity environments cause a decrease in serum testosterone levels in men [129,130]. As the site of sperm production and storage, the normal functioning of the testes is important for male reproductive health. It has been shown that high levels of ROS can cause significant damage to the structure and function of the testes by triggering ferroptosis [131]. In addition to this, there are several male reproductive disorders associated with high levels of the ROS. OS levels are significantly elevated in patients with varicocele and reduced semen quality in patients [132]. Some inflammatory reproductive tract infections in men (e.g., prostatitis, epididymitis, etc.) can cause high levels of ROS production, thus impairing male reproductive function even more [133]. In conclusion, space-based male reproductive health faces significant challenges due to the high levels of ROS triggered by microgravity environments, which may lead to a variety of male diseases or even cause male infertility.

#### 3.5.3. Microgravity-Induced ROS on Embryonic Development

The microgravity-induced ROS has an effect on embryonic development. Similar to sperm and eggs, embryos in the presence of high levels of ROS can also affect embryonic development [134], which may ultimately lead to embryonic developmental arrest. For preimplantation embryos, the effects of OS are particularly significant and may lead to delayed embryonic development, reduced blastocyst formation, and implantation failure [135]. At the post-implantation stage, oxidative damage may affect organ formation and tissue differentiation of the embryo, increasing the risk of miscarriage and fetal malformations. Embryos in microgravity are also affected due to the oxidative stress caused by microgravity. Mouse embryonic stem cells (mESC) are often used as a model for embryonic stem cell research, and Ran et al. [63] showed SMG can increased ROS production in mESC. Microgravity also induces apoptosis in mESC [13]. This suggests that we should investigate how microgravity-induced OS affects embryonic stem cell apoptosis. Furthermore, microgravity influences the differentiation of embryonic stem cells [14]. Early embryo lethality is increased due to microgravity, and blastocyst formation is also affected in the space environment [15,136]. Therefore, focusing on microgravity-induced oxidative damage in embryos is also very meaningful for the study of embryonic development in space.

## 4. Detection of Oxidative Stress Using Fluorescence Probes

In order to better investigate the effects of cellular OS in microgravity, it is necessary to develop monitoring methods for ROS that have a real-time monitoring effect, high sensitivity, high specificity, and easy operation. Among the various methods, various fluorescent probes stand out as “tracers” for ROS tracking. Fluorescent probes are fluorescent molecules consisting of a fluorophore, a recognition site, and a linker. Fluorescent probes can emit fluorescence when irradiated with specific wavelengths of excitation light to label specific organelles or molecules in living cells, and can be imaged for long periods of time to reflect the dynamic information within the cell. The high specificity of fluorescent probes makes it possible to detect some specific molecules, including various molecules of the ROS.

### 4.1. Fluorescent Probes for the Detection of ROS

**Figure 4:**
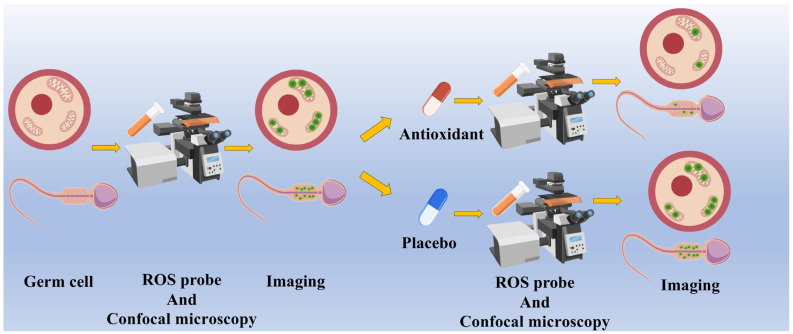
ROS fluorescent probes for oocyte or sperm imaging. The ROS fluorescent probe was added to the culture medium of the oocyte or sperm and imaged with a confocal microscope. A lot of green fluorescence can be observed on the oocyte or sperm, which represents where the ROS are located. The number and intensity of the fluorophores can indicate the degree of OS. A significant reduction of OS in the oocyte or sperm can be observed after treatment with antioxidants.

In recent years, a number of fluorescent probes with high sensitivity and good spatial, as well as temporal resolution, have emerged as a major tool for OS studies (Figure 4).

#### 4.1.1. DCFH-DA

DCFH-DA is a widely used ROS fluorescent probe. Its chemical name is 2′,7′-dichlorodihydrofluorescein diacetate [137], and DCFH-DA can detect a variety of intracellular ROS, including H_2_O_2_, O_2_^−^, OH, etc. DCFH-DA can freely cross through the cell membrane of the cell, and then it will be hydrolyzed by the esterase of the cell to convert two ester groups into H_2_DCF. Since H_2_DCF can no longer cross the cell membrane, it remains inside the cell. H_2_DCF can oxidize with the ROS generated by cells, oxidizing to DCF and releasing fluorescent signals [138]. The benefit of DCFH-DA is its ability to remain in the target cell for an extended period of time while maintaining a steady fluorescence signal.

#### 4.1.2. MitoSOX

MitoSOX is also a commonly used fluorescent probe for the detection of peroxides, but unlike DCFH-DA, MitoSOX targets cellular mitochondria and can only detect the ROS in mitochondria [139]. The most common and widely used type of MitoSOX is MitoSOX Red, a dihydroethidium derivative that carries a triphenylphosphine cation, which helps the MitoSOX Red enter the cell and target the mitochondria [140,141,142]. MitoSOX Red that enters the mitochondria specifically recognizes O_2_^−^ and is activated to fluoresce in red. MitoSOX is often used for the diseases caused by mitochondrial OS, such as degenerative diseases. In addition, the mitochondrial ROS are important cellular signals, and MitoSOX has also been used to study the role of peroxides in cellular signaling pathways.

#### 4.1.3. CellROX

CellROX is a new type of probe for intracellular ROS detection. CellROX fluoresces very weakly or hardly fluoresces before it enters the cell (in the reduced state), but when it enters the cell with elevated levels of intracellular ROS, CellROX is oxidized and generates a strong fluorescent signal that labels various superoxides, including H_2_O_2_, O_2_^−^, HOOO−, ONOO−, etc. [143,144]. CellROX can be classified into three types according to the fluorescence color of excitation: CellROX Green Reagent, CellROX Deep Red Reagent, and CellROX Orange Reagent. CellROX has a number of advantages compared to other ROS fluorescent probes: its triple-color product allows flexibility in designing experiments, such as co-localization staining, and CellROX is more photostable.

### 4.2. ROS Fluorescent Probes in Germ Cell Research

OS in germ cells has opened up a new “battlefield” for fluorescent probes, and various ROS fluorescent probes have made a great impact in the field of reproductive health research (Table 1). Recently, Escada-Rebelo et al. compared different fluorescent probes for the detection of sperm ROS [145]. The results show that, for human sperm, MitoSOX Red and DHE are more specific for O_2_^−^, while CellROX Orange Reagent, Redox Sensor “Red CC-1”, and MitoPY1 are more sensitive to H_2_O_2_. This provides guidance for the specific detection of different ROS in human spermatozoa by different fluorescent probes. Fluorescent probes for evaluating the oocyte quality and developmental potential are commonly used as DCFH-DA probes, and in recent years a number of new fluorescent probes have emerged for detecting the oocyte quality. Javvaji et al. [146] applied nitroblue tetrazolium salt (NBT) as a fluorescent probe and used a new method for detecting intra-ovarian ROS in the oocytes, granulosa cells, and embryos using NBT staining and bright-field microscopy. This method produces a very stable blue methanol precipitate, less susceptible to photobleaching and redox reactions than the DCFH-DA probe, and has a higher specificity. Dong et al. [147] introduced a dual-activated H_2_O_2_-responsive AIE fluorescent probe, b-PyTPA, which reacted with H_2_O_2_ to generate PyTPA with enhanced hydrophobicity, and this increase in hydrophobicity promoted the aggregation of PyTPA molecules, which led to a significant enhancement of fluorescence intensity due to the aggregation-induced luminescence property of AIE. It can be used as a novel ROS fluorescent probe to detect the degree of OS in the oocytes in order to judge the quality of the oocytes. In conclusion, various ROS fluorescent probes can reflect the degree of OS in germ cells by making the ROS “visible” in germ cells, which makes the ROS fluorescent probes become a new strategy for the detection of germ cell quality to protect human reproductive health.

### 4.3. Limitations and Challenges of Fluorescent Probes to Detect ROS in Germ Cells in Microgravity

Despite the evident advantages of fluorescent probes, such as their capacity for visualization and intuitiveness, there are still some limitations and challenges in applying them to detect ROS production by germ cells in microgravity in the future. First, a true microgravity environment can only be realized in space. If fluorescent probes are to be used in space to target the ROS produced by OS in germ cells, there will be many difficulties. For example, in microgravity, adherent cells may be suspended and cause abnormal adherence [154], and the properties of the medium and the fluorescent probe as a fluid are different in microgravity from those on the ground, which may make it difficult to show the real situation of ROS production. While a microfluidic chip can realize operations such as fixation of fluids and quantitative addition of fluorescent probes, building a set of microfluidic system is a feasible strategy for the application of fluorescent probes in space [155]. It is very expensive to bring something into space, and fluorescent probes often need to be washed in order to reduce the interference of background signals. The use of wash-free probes will facilitate this space experiment, as the cost of carrying washing solutions into space will inevitably increase. Many AIE fluorescent probes have this advantage, and will become a preferred class of probes for detecting the ROS in germ cells in microgravity. Recently, a study showed that ASCPB can be used as an AIE probe and utilized for fluorescence imaging of the ROS in microgravity with the advantage of being wash-free [156]. Long-term results are often desired for research in space, so the properties of the fluorescent probe itself should be able to meet the needs of long-time observation. This necessitates probes that exhibit excellent photostability and are resistant to photobleaching, which leads to the weakening of the fluorescence signal. Probes, such as CellROX Orange, possess strong photostability and are resistant to photobleaching, enabling prolonged imaging [157]. To investigate the effects of microgravity on germ cell ROS more deeply and precisely, fluorescent probes need to be sensitive enough to detect very small changes in ROS concentration and convert them into fluorescent signals, which requires researchers to develop ROS fluorescent probes with higher sensitivity. In addition, different fluorescent probes target the different types of ROS, and the ROS produced by germ cells in microgravity are mostly H_2_O_2_ and O_2_^−^. If we want to study the production of a specific ROS by germ cells, we need fluorescent probes with excellent specificity, which also need to be further researched and developed. Microgravity induced various reactions on germ cells, such as cell membrane permeability, mitochondrial structure changes, etc., which may also affect the localization and function of fluorescent probes, and also need to be paid attention to. In summary, although the application of fluorescent probes for the detection of ROS in germ cells under microgravity is promising, many challenges still need to be overcome. Future studies should focus on the optimization of probe performance and the adaptation of germ cell responses in microgravity environments to promote the application of ROS fluorescent probes in space biology research.

## 5. Protective Countermeasures for Oxidative Stress in Microgravity

It has been previously addressed how OS remodels germ cells in a microgravity setting. Under such circumstances, elevated ROS levels have serious potential reproductive risks. To combat any oxidative damage in space, antioxidant measures must be used in addition to activating the body’s natural oxidative homeostasis system.

### 5.1. Antioxidant Supplementation

#### 5.1.1. Vitamin C

As a water-soluble vitamin, vitamin C is well known for its antioxidant effects. The reducing properties of vitamin C facilitate the elimination of the toxicity of harmful oxygen radicals through reduction reactions. Vitamin C can inhibit ovarian senescence by scavenging the ROS and attenuating DNA damage, improving the quality of the oocytes [158]. Vitamin C might increase progesterone secretion in women with luteinizing hormone deficiency due to OS [159,160]. For male reproductive health, studies have demonstrated a correlation between decreased levels of vitamin C and various male reproductive disorders, including asthenospermia and varicocele [161]. Furthermore, the significance of vitamin C for the fetus is noteworthy. Studies have demonstrated that maternal vitamin C deficiency during pregnancy does not affect fetal development in the womb. However, it has been observed to have a substantial impact on the developing adult fetus, resulting in a reduced number of germ cells, delayed meiosis, and diminished fecundity in female offspring [162]. This finding suggests a potential link between the antioxidant effects of vitamin C and the reproductive health of female offspring.

#### 5.1.2. Vitamin E

Vitamin E, also known as tocopherol, serves as a very valuable antioxidant vitamin. Vitamin E protects the cell membranes of germ cells and mitochondrial membranes by protecting the unsaturated fatty acids in cell membranes from oxidation, and also protects germ cells by scavenging free radicals and preventing lipid peroxidation from occurring [163]. For women, vitamin E protects the oocytes, safeguards ovarian health, and reduces OS in the corpus luteum [158]. For men, vitamin E has been shown in several studies to increase sperm concentration and viability and reduce the probability of sperm malformations. Vitamin E in combination with other antioxidants, such as vitamin C and selenium, may also enhance antioxidant effects and improve sperm quality [164].

#### 5.1.3. Coenzyme Q10

Coenzyme Q10 (CoQ10), also known as ubiquinone, is a coenzyme involved in the process of mitochondrial aerobic respiration in eukaryotes. CoQ10 has been shown to have the capacity to directly scavenge free radicals [165]. Additionally, it has been observed to enhance a variety of in vivo antioxidant enzymes, such as SOD and CAT, in response to OS [166]. In germ cells, the antioxidant effects of CoQ10 are also essential. Aging-induced OS has been identified as a primary contributor to the decline in ovarian function in women, and CoQ10 has been observed to reverse the decline in the oocyte number and quality in aged mice [167]. Xu et al. [168] found, by meta-analysis, that CoQ10 treatment led to an increase in the oocyte count, fertilization success, and embryo quality in patients with poor ovarian response. CoQ10 has been shown to enhance sperm quality and viability and to reverse the formation of malformed sperm in males [169]. CoQ10 can also reduce DNA fragmentation and ROS markers in sperm. These findings collectively suggest that CoQ10 exerts its beneficial effects on the quality of germ cells through its antioxidant mechanisms.

#### 5.1.4. Controversy over Conventional Antioxidants

Apart from vitamins and CoQ10, a range of antioxidants, including quercetin, curcumin, and resveratrol, have given germ cells additional strategies for resisting OS (Table 2). Although many studies have shown that conventional antioxidants are very effective for germ cells against OS, this is also controversial. Steiner et al. [170] conducted a study with antioxidants in men with substandard sperm quality, where patients in the treatment group were given multiple antioxidants daily. Surprisingly, after several months of treatment for all patients, those in the antioxidant group did not differ significantly from the placebo group in terms of sperm morphology, viability, or DNA fragmentation. Not coincidentally, Kaitsas et al. [171] noted that, although many studies have shown that antioxidant supplements can improve male fertility, there are also studies that show inconsistent results, and even some studies that did not find a significant positive effect of antioxidants on male fertility. In addition, in patients undergoing in vitro fertilization (IVF), the percentage of mature oocytes and the number of first-degree embryos is instead reduced when the blood levels of alpha-tocopherol, which is an active form of vitamin E, are too high [158].

In response to these controversies, several possible reasons have been postulated. Firstly, using antioxidants separately might not have the same effects as using them in combination. It is also possible that some of the antioxidants used in combination may combine their antioxidant effects, while others may inhibit each other. Secondly, the dosage of antioxidants seems to be crucial for producing a therapeutic impact, highlighting the need to determine the ideal dosage for additional research. This variability may be attributable to various factors, including the study design, the sample size, and the patient selection. Future studies should draw on the results of earlier antioxidant research and carry out more precise trials to better meet human demands for reproductive research in space in order to solve the OS caused by the microgravity environment.

### 5.2. Gene Editing

Gene editing is the technique of modifying specific genes in the genome, which can lead to alterations in the DNA sequence, which in turn leads to changes in the transcription of genes and the expression of proteins, and ultimately to alterations in the phenotypic characteristics of organisms. Some studies have shown that gene editing technology can facilitate antioxidant measures. Yan et al. [180] used gene editing technologies to handle human embryonic stem cells and increase FOXO3 expression. The differentiated vascular cells were discovered to have a greater capacity for antioxidants. Another study showed that MSCs showed enhanced antioxidant capacity by removing the Keap gene through gene editing [181]. Huang et al. [182] established a germ cell-specific transgenic mouse model based on the antioxidant gene LanCL1. Their study found that LanCL1 is a male germ cell-specific marker, and that specific overexpression of LanCL1 in spermatocytes resists oxidative damage to spermatocytes. This opens up one more avenue of research through gene editing for the link between OS and male fertility. Through gene editing, it is even theoretically possible to alter germ cell genes related to antioxidant enzymes for the purpose of improving antioxidant capacity. For example, in the future, the expression of antioxidant-related genes could be directly enhanced by gene editing tools, such as CRISPR-Cas9. For example, the Nrf2 gene can be edited to activate the expression of antioxidant enzymes, such as SOD, and the ability of germ cells to scavenge ROS under microgravity can also be enhanced by gene knock-in or overexpression.

### 5.3. Exercise and Physical Intervention

Exercise is also an effective way to reduce OS. Exercise can enhance the antioxidant capacity of mitochondria and even improve mitochondrial dysfunction [183]. Exercise also significantly increases the activity of antioxidant enzymes, such as SOD, which directly scavenges excess free radicals [184]. Endurance training enhances the antioxidant capacity of the heart to protect itself [185]. Therefore, using exercise to reduce OS in germ cells under microgravity is also one of the feasible strategies.

Another way to combat microgravity that deserves attention is to simulate Earth’s gravity in space to eliminate the OS caused by SMG. The Multiple Artificial-gravity Research System (MARS) is an experimental platform developed by the Japan Aerospace Exploration Agency (JAXA). MARS can simulate 1g of Earth’s gravity in space. Several space studies have shown that the artificial gravity brought by MARS can play a key role in combating the effects of microgravity [186,187,188]. Although there are no experiments in which MARS was used to combat OS in germ cells under microgravity, artificial-gravity may become an important method to combat the OS caused by microgravity.

Each of these four methods has advantages and disadvantages, and their feasibility for application in space varies. We have summarized them below (Table 3).

## 6. Conclusions and Perspectives

In summary, OS induced by microgravity poses a serious threat to the health of both somatic cells and germ cells. We discuss the molecular mechanisms by which microgravity induces OS through multiple pathways, leading to widespread effects on cells. Although many biological mechanisms are expounded based on somatic cells, many mechanisms are common because somatic cells and germ cells have many consistencies at the cellular level. However, due to the heritability of germ cells, their normal growth and differentiation in space are of even greater importance. Therefore, in this article, we have extensively discussed the impact of the oxidative stress caused by microgravity on germ cells.

Fluorescent probes have been identified as effective tools for detecting intracellular ROS levels, although current fluorescent probes have certain limitations. Additionally, we propose a series of protective measures, which may hold potential value in mitigating oxidative damage.

To enhance the detection and mitigation of OS in reproductive cells induced by microgravity, the following research directions should be considered. First, the optimization of existing indicators for detecting the ROS, such as related enzyme activity levels or OS products like MDA, is necessary. However, these methods have limitations in detecting specific ROS types and can only provide an overview of the total ROS levels. Additionally, current methods exhibit limited sensitivity, often failing to detect subtle changes in the ROS levels. Therefore, these assays cannot accurately reflect real-time ROS fluctuations. Consequently, the development of novel ROS detection technologies, such as ROS fluorescent probes, is required. These probes should possess higher specificity and sensitivity and enable real-time monitoring, thereby providing more accurate detection results. Second, the development and evaluation of new antioxidants can serve as a means to mitigate the effects of OS. Some natural products have demonstrated excellent antioxidant effects. For example, curcumin, icariin, and lycopene have been observed to enhance the activity of antioxidant enzymes by activating the Nrf2-related pathway, thereby promoting cell survival under OS conditions. Furthermore, there is a need to further investigate the signaling pathways involved in OS in reproductive cells under microgravity conditions. It is also important to identify which activators and inhibitors can activate or inhibit these pathways to alleviate OS. Customized antioxidant supplementation plans can be developed based on individual genetic profiles. For instance, individuals carrying SOD2 mutations should consider increasing their intake of naturally SOD-rich foods, such as apples and broccoli. Finally, since actual microgravity experiments are conducted in space, it is crucial to encourage data sharing among the space station and national space agencies to establish a global microgravity reproductive medicine database. It is believed that the promotion of a variety of measures will be beneficial to all of humanity in addressing the future of reproductive health in space.

## Figures and Tables

**Table 1:** Fluorescent probes for detecting ROS in germ cells.

Fluorescent Probe	Target	Excitation/Emission Wavelength (nm)	Sensitivity	Specificity	Usability	Applications on Germ Cells	Ref
DCFDA	H_2_O_2_, O_2_, •OH, etc.	488/525	Moderate	Moderate	Wide range of applications. Can be used with fluorescence microscopy or confocal microscopy, etc.	Study the effect of heparin on ROS activity in goat spermatozoa.	[148]
MitoSOX	O_2_^−^	510/580	High	High	One of the preferred tools for the detection of mitochondrial superoxide in living cells. Can be used with fluorescence microscope or confocal microscope.	Compare ROS production in the oocytes of mice on a high-fat diet and mice supplemented with nicotinamide riboside (NR).	[149]
CellROX Orange	H_2_O_2_, O_2_^−^, HOO^−^, ONOO^−^, etc.	545/565	Moderate	Moderate	Compatible with live cells and easy to operate, can be detected by fluorescence microscope, flow cytometer, and so on.	Semen parameters and ROS levels were found to have a positive correlation.	[150]
Nitroblue tetrazolium (NBT)	O_2_^−^	/	Moderate	Moderate	Easy to use, can be detected by bright-field microscopy, but the results are relatively crude.	Matured COC and embryos were stained with NBT to detect and quantify intracellular ROS.	[145]
b-PyTPA	H_2_O_2_	488/669	High	High	Dual activation of oxidative stress response, can be detected by confocal microscopy.	This Dual-Activated H_2_O_2_-Responsive AIE probe can used for the detection of the ROS in oocytes, and enable the assessment of their quality.	[146]
AIE-FR-TPP	H_2_O_2_, O_2_^−^, •OH, etc.	488/662–737	High	High	Targets mitochondrial ROS with low working concentration and good biocompatibility. Can be detected by confocal laser microscopy.	AIE-FR-TPP probe is used for mitochondrial imaging, photodynamic therapy, and visualizing the therapeutic process in zebrafish embryos.	[151]
PeroxyBODIPY-1(PB1)	H_2_O_2_	490/509	High	High	Cell-permeable, can be detected by confocal microscopy.	PB1 is able to detect H_2_O_2_ in denuded bovine oocytes.	[152]
DHR123	H_2_O_2_, O_2_^−^, ONOO^−^, NO	488/530	High	Moderate	Targets mitochondria, but prolonged light exposure leads to fluorescence quenching, can be detected by flow cytometer or fluorescence microscope.	DHR123 can afford a simple method to measure OS in human spermatozoa.	[153]

**Table 2:** Antioxidants reduce oxidative stress in germ cells.

Antioxidants	Cell Type	Findings	Ref
CoQ10	4–6 week-old female mice oocyte	CoQ10 can inhibit the OS caused by aging and inhibit the apoptosis of the oocytes by reducing the levels of peroxide and DNA damage in the oocytes.	[172]
Vitamin C	Mice 2-cell embryos	Vitamin C alleviates embryonic OS from juglone, reduces abnormal mitochondrial potentials and epigenetic modifications, and reduces excess intracellular ROS levels, DNA damage, and embryo apoptosis.	[173]
Vitamin E Nanoemulsion	Manchega rams’ sperm	Vitamin E nanoemulsion gives sperm greater motility than free vitamin E and also protects against the harmful effects of free radicals and lipid peroxidation caused by OS.	[174]
Resveratrol	Female mice MII oocytes	Resveratrol maintains homologous mitochondrial distribution in postovulatory aging (POA) oocytes and has an antiapoptotic effect on the POA oocytes, and also protects against H3K9me2 methylation deletion, protects epigenetic modifications in the POA oocytes, and improves subsequent development of blastocysts.	[175]
Curcumin	Porcine granulosa cells (GCs)	Curcumin attenuates afb1-induced growth inhibition and mitigates mitochondrial dysfunction, thereby reducing OS in porcine GCs.	[176]
Salidroside	Female mice MII oocytes	Salidroside can decreases the malformation rate and recovers mitochondrial dysfunction, relieves the OS caused by aging after ovulation, promotes protective autophagy of the senescent oocytes by activating MAPK pathway.	[177]
Lycopene (LYC)	Bovine sperm	LYC showed significant ROS clearance and antioxidant properties, which could improve sperm motion parameters and mitochondrial activity	[178]
Quercetin	Goat sperm and zygotes.	Quercetin can reduce the MDA and ROS levels of oxidized sperm, maintain sperm motility and various characteristics, and promote zygote to reduce peroxide under OS, maintain mitochondrial function and ensure embryo quality.	[179]

**Table 3:** Comparison among different antioxidant pathways.

Methods	Relative Effectiveness	Feasibility in Space	Possible Side Effects.
Antioxidant Supplementation	Moderate; Conventional antioxidants are effective in scavenging free radicals, attenuating oxidative stress, and improving oocyte quality, but they cannot completely eliminate the oxidative damage caused by microgravity.	High; Traditional antioxidants have simpler preservation and use conditions, are easy to carry and use in the space environment, and are relatively inexpensive.	Single antioxidants may have limited antioxidant capacity, may be potentially toxic at high doses, and there may be interactions between different antioxidants that can affect effectiveness.
Gene Editing	High; Gene editing can accurately edit genes related with antioxidants to enhance the antioxidant capacity of stem cells from the root, with long-lasting and stable effects.	Low; The application of gene editing technology in the space environment faces many challenges, such as high equipment requirements, complex operation, and the risk of damage to cells, etc., and requires the support of specialized technicians and equipment, and the application in space is still in the preliminary exploration stage.	It may lead to gene mutation, off-target effects, etc., affecting the normal physiological function and genetic stability of germ cells, and is also ethically controversial.
Exercise	Moderate; Exercise enhances the body’s antioxidant capacity, promotes blood circulation, improves the nutrient supply and metabolic environment of germ cells, and has a positive effect on reducing oxidative stress under microgravity.	Moderate; In the space environment, exercise requires specialized equipment and space and is limited by microgravity conditions, with limited exercise modalities and intensity, but can be achieved through the design of rational exercise programs and the use of special exercise equipment.	Excessive exercise may lead to physical fatigue, injury, and increase the production of free radicals, which in turn aggravates oxidative stress, and an appropriate exercise program needs to be developed on an individual basis.
Physical Intervention	Theoretically the highest; 1 g mimicry restores cytomechanical signaling and maintains mitochondrial membrane potential and ROS homeostasis	Moderate; Artificial gravity devices are typically large, heavy, and energy intensive, placing extreme demands on the design and operation of spacecraft.	The effects of long-term exposure to artificial gravity on the body need to be further studied.

## Data Availability

Data sharing is not applicable to this article.
